# Monte carlo assessment of advanced shielding materials for space‐efficient radiotherapy vault design

**DOI:** 10.1002/acm2.70728

**Published:** 2026-07-30

**Authors:** Beechui Koo, Richard Xu, Morgan Glennie, Jungwook Shin, Karl Farrey, Siyong Kim, James J. Sohn

**Affiliations:** ^1^ Data Science Institute University of Chicago Chicago Illinois USA; ^2^ Department of Radiation Medicine University of Wisconsin‐Madison Medison Wisconsin USA; ^3^ Division of Cancer Epidemiology and Genetics National Cancer Institute National Institutes of Health Rockville Maryland USA; ^4^ Department of Radiation Oncology Virginia Commonwealth University Richmond Virginia USA

**Keywords:** Monte Carlo simulation, NCRP 151, radiation shielding design, tenth‐value layer, vault optimization

## Abstract

**Background:**

Conventional radiotherapy vault shielding typically employs 2 to 3 meters of ordinary concrete, consistent with NCRP Report 151 reference data. While NCRP 151 specifies dose‐based design goals rather than mandating particular materials, the practical default of concrete results in vault footprints of 150 to 200 m^2^ per linac, which can constrain treatment capacity within fixed departmental space. Recent advances in high‐density composite shielding materials offer the potential to achieve the same dose‐based design goals with reduced barrier thickness.

**Purpose:**

In this study, we investigate advanced shielding materials, including specialty concretes and high‐density polymer composites, as potential alternatives to conventional concrete barriers, aiming to demonstrate that modern materials could safely enable more compact vault designs while maintaining regulatory compliance.

**Methods:**

TOPAS Monte Carlo simulations evaluated a 6 MV and an 18 MV linear accelerator (LINAC) vault comparing conventional concrete against five candidate shielding materials. We modeled a typical 4‐vault radiotherapy department to demonstrate potential capacity increase. Simulations calculated linear attenuation coefficients (μ), tenth‐value layers (TVLs), and dose distributions at regulatory measurement points. Spatial analyses projected the clinical impact of implementing optimized shielding.

**Results:**

While concrete required 165.8 m2 floor area per vault, candidate materials achieved 17%–32% reductions for 6 MV beams and 22%–34% reductions for 18 MV beams. Specifically, Steel‐magnetite and Datolite‐Galena concretes showed great space‐saving potentials with footprint reductions of 28% and 31% for 18 MV beams, respectively, while maintaining refined doses at 2.40 × 10−2 and 2.42 × 10−2 mSv/week, well below the 0.1 mSv/week NCRP limit.

**Conclusion:**

This study demonstrates that high‐density composite shielding may reduce vault footprint while maintaining dose levels below established shielding design limits. These findings suggest that systematic evaluation of alternative shielding materials may provide useful insights for space‐efficient radiotherapy vault design while remaining within established dose‐based shielding frameworks.

## 1 INTRODUCTION

High‐energy medical linear accelerators require substantial radiation shielding to meet regulatory dose limits for workers and the public. National Council on Radiation Protection and Measurements (NCRP) Report 151 recommends primary barrier thicknesses of 2.0–3.0 meters of ordinary concrete (density ∼2.3 g/cm^3^) for conventional LINAC vaults.^1^ These requirements result in treatment vaults consuming 150–200 m^2^ per LINAC, significantly limiting the number of treatment units that can be accommodated within hospital radiation oncology departments. Modern accelerator designs like self‐shielded systems and alternative shielding materials motivate renewed evaluation of material selection and barrier‐thickness efficiency within established dose‐based shielding design frameworks.^2^ To provide the most stringent test of alternative shielding materials, the present study models an 18 MV beam, representing the upper bound of clinical photon energies and associated shielding demands.

The attenuation of high‐energy photons in matter follows exponential decay characterized by the linear attenuation coefficient (μ), with barrier effectiveness typically expressed as TVLs. For 18 MV beams, ordinary concrete exhibits μ ≈ 0.075 cm^−1^ and TVL ≈ 31 cm.^3^ Lead, while offering superior attenuation (μ ≈ 0.5 cm^−1^), presents toxicity concerns and poor neutron shielding properties due to (γ,n) reactions above 7 MeV.^4^ The ideal shielding material would combine high photon attenuation, neutron moderation capability, structural integrity, and non‐toxicity.

Recent advances in shielding materials offer promising alternatives. The incorporation of high‐Z additives, including nanoparticles, mineral aggregates, and metallic components, into polymer or concrete matrices can significantly enhance attenuation properties while maintaining structural integrity. Kazemi et al. demonstrated that polyvinyl alcohol containing 50% by weight nano‐sized WO_3_ achieved mass attenuation coefficients 40% higher than equivalent micro‐particle composites, attributed to uniform dispersion minimizing radiation streaming paths.^5^ Similarly, steel‐magnetite concrete (density ∼3.5 g/cm^3^) and bismuth oxide‐loaded concrete have shown TVL reductions of 50%–60% compared to ordinary concrete while maintaining neutron moderation through their hydrogen content.^6^ Monte Carlo simulations by Rosenstrom et al. indicated that optimized multi‐layer configurations could achieve equivalent shielding in 70% of the thickness required for concrete.^7^

In space‐constrained radiation oncology departments, reducing shielding‐related wall thickness may improve facility layout flexibility and treatment‐room planning. With global radiotherapy demand projected to increase by 50% by 2035,^8^ optimizing vault design becomes critical for healthcare access. In this study, we employ TOPAS Monte Carlo simulations to systematically evaluate five candidate shielding materials against conventional concrete for a 6 MV and an 18 MV LINAC vault. Our objectives are to: (1) determine material‐specific attenuation parameters (μ, TVL_1_, TVL_2_), (2) validate dose distributions meet NCRP‐151 limits, and (3) quantify potential space savings and capacity gains achievable through advanced shielding materials.

## 2 METHODS AND MATERIALS

### 2.1 Monte carlo simulation for linear attenuation coefficient and TVL of materials

**FIGURE 1:**
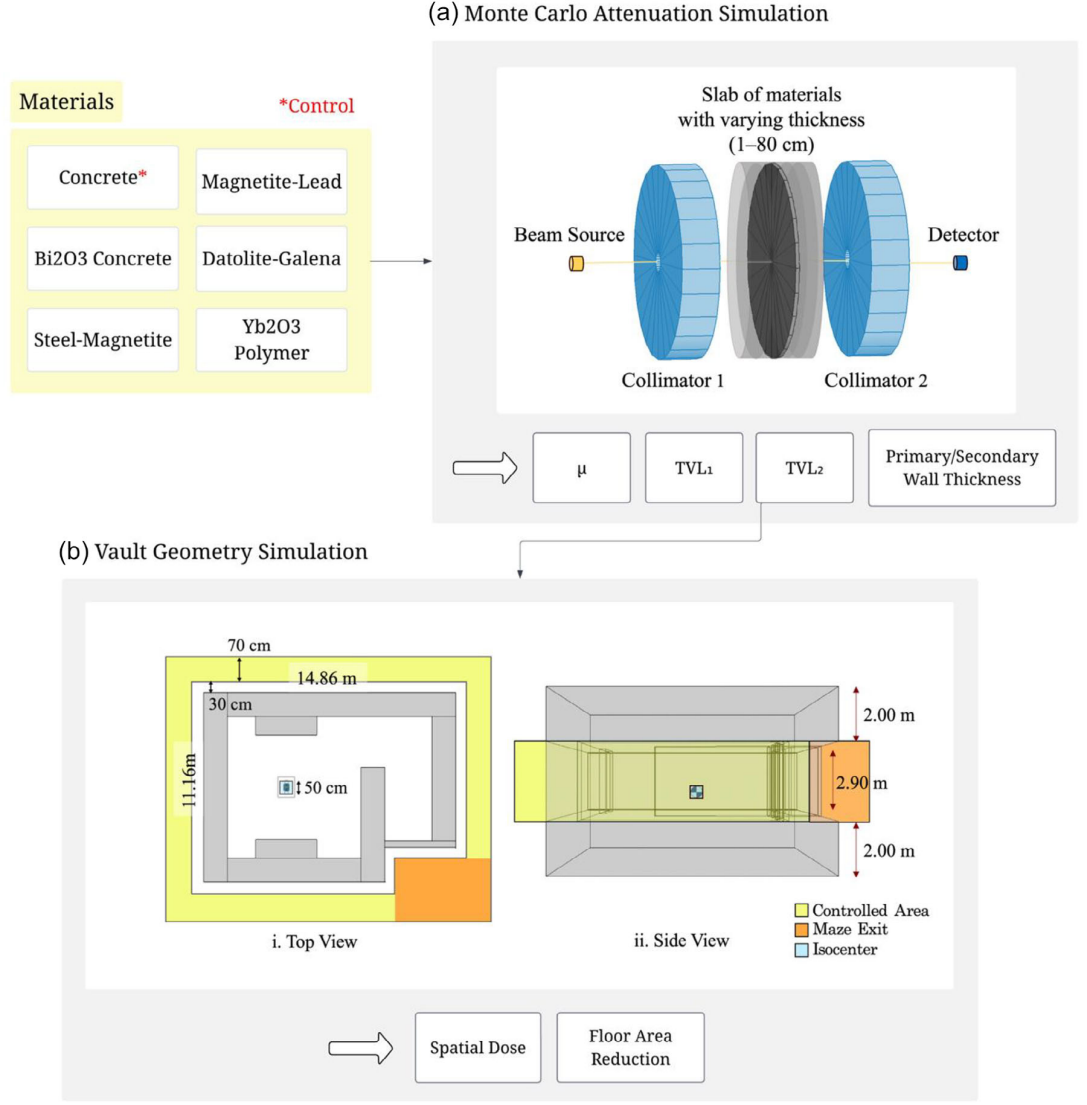
Schematic overview of the experimental workflow. Each of the five selected materials, along with conventional concrete, undergoes (a) Monte Carlo–based attenuation modeling using narrow‐beam geometry and subsequently implemented in a (b) full LINAC vault model to evaluate dose compliance and spatial efficiency.

All simulations were performed using TOPAS (version 3.7) Monte Carlo code, which serves as a wrapper for the Geant4 toolkit and is widely used for detailed radiation transport modeling in medical physics.^9^ In this study, the geometry shown in Figure 1a was designed to model a narrow beam for calculating the attenuation coefficient of each material. Two circular lead collimators, each 40 cm thick with an outer radius of 100 cm, were positioned on either side of the test slab. The beam source, collimators, slab, and detector were spaced 100 cm apart. The aperture size was chosen to approximate narrow‐beam geometry, minimizing the contribution of scattered photons to the transmitted beam. Siemens Oncor 6 MV and 18 MV linac photon beams were simulated, incorporating directional Russian rouletting and bremsstrahlung splitting with a split number of 1:300 to reduce variance and computation time. The narrow‐beam setup ensures that the photons reaching the detector retain their original energy and are not influenced by scattering or secondary interactions. The materials investigated are listed in Table 1, along with their elemental compositions and densities.^6^, ^10^, ^11^, ^12^, ^13^, ^14^, ^15^ For each material, slab thicknesses at the center of the beam were varied across 19 values: increments of 1 cm from 1–10 cm, 5 cm from 10–40 cm, 10 cm from 40–60 cm, and a final value at 80 cm. A baseline fluence was also measured with no slab present. Each configuration was simulated three times with independent random seeds to estimate statistical uncertainty, confirming that all dose tallies achieved relative statistical uncertainties below 5%. The detected beam intensity depends on the initial beam intensity and the linear attenuation coefficient of the intervening material, as described by Lambert's law:^16^

$$I=I_{0}\;e^{-\mu t},$$

where $I_{0}$ is the incident intensity, μ is the linear attenuation coefficient, and t is the material thickness. For each material studied, the attenuation coefficient was derived from the differential form of this law:^16^

$$\;\lim_{\Delta t\to 0}\frac{\Delta I/I}{\Delta t}=-\mu ,$$

where Δt represents the incremental thickness of the material and ΔI/I is the fractional reduction in beam intensity over that increment. This relationship emphasizes that the linear attenuation coefficient quantifies the proportional decrease in intensity per unit thickness, independent of the beam's absolute intensity.

**TABLE 1:** Elemental composition and density (g/cm3) of the materials used in this study.

Element	Concrete	Steel‐Magnetite	Bi_2_O_3_ Concrete	Magnetite‐Lead	Datolite‐Galena	Yb_2_O_3_ Polymer
Density (g/cm^3^)	2.30	5.106	3.30	4.60	5.10	6.90
H	0.006	0.0051	0.012	0.006		0.08
O	0.532	0.1571	0.3473	0.247	0.29	0.2643
Si	0.337	0.0266	0.15	0.015	0.04	–
Ca	0.06	0.0394	0.072	0.01	0.08	–
Al	0.05	0.0065	0.042	–	–	–
Fe	0.015	0.7577	0.018	0.442	–	–
Mg	–	0.0057	–	–	–	–
S	–	0.0006	–	–	0.03	–
C	–	–	–	–	–	0.48
P	–	0.0008	–	–	–	–
Mn	–	–	–	–	–	–
Pb	–	–	–	0.28	0.55	–
B	–	–	–	–	0.01	–
Bi	–	–	0.3587	–	–	–
Yb	–	–	–	–	–	0.1757
W	–	–	–	–	–	–

From the simulated transmission data, the first tenth‐value layer (TVL_1_) was defined as the thickness at which the transmitted intensity decreased to 10% of its initial value. The second tenth‐value layer ($\mathrm{TV}L_{2}$) was defined as the additional thickness required to reduce the transmitted intensity from 10% to 1%. Mathematically, these values were calculated as:

$$\mathrm{TV}\;L_{1}=\frac{log_{10}\left(I_{0}/I_{1}\right)}{\mu_{1}}\;\;\mathrm{and}\;\mathrm{TV}\;L_{2}=\frac{log_{10}\left(I_{1}/I_{2}\right)}{\mu_{2}}$$

where I_1_ and I_2_ represent the intensities corresponding to 0.1 I_0_ and 0.01 I_0_, respectively.

The equilibrium tenth‐value layer (TVL_e_) was obtained from the subsequent portion of the attenuation curve after the buildup region, reflecting the slope of the linear region in a semilogarithmic plot of transmission versus thickness:

$$\;\log_{10}\left(\frac{I}{I_{0}}\right)=Intercept-\frac{Thickness}{TVL_{e}}$$

where the slope of the fitted region corresponds to: $TV\;L_{e}=-\frac{1}{\mathrm{slope}}$.

### 2.2 Barrier thickness determination and refinement

#### 2.2.1 Preliminary thickness estimation

The materials were simulated within a realistic LINAC vault maze geometry to evaluate their shielding effectiveness and to assess potential secondary radiation effects. Based on the linear attenuation coefficients and TVL data obtained from Section 2.1, the required thicknesses of both primary and secondary barriers were first calculated for each candidate material to achieve the same transmission factor as standard NCRP‐151–compliant concrete.

To enable a consistent comparison between conventional NCRP‐151 compliant concrete and the candidate shielding materials, the required barrier thickness for each material was determined based on transmission equivalence. The objective was to preserve the same transmission factor while accounting for material‐dependent attenuation properties.

The required transmission factor B for a given barrier was defined according to standard shielding formalism:

$$B=\frac{P\cdot d^{2}}{W\cdot U\cdot T}$$

where P represents the design dose limit beyond the barrier, d is the distance from the radiation source to the point of interest, W denotes the workload, U is the use factor, and T is the occupancy factor. For secondary barriers, leakage and patient‐scatter contributions were incorporated following NCRP‐151 methodology.

The number of TVLs required to achieve the target transmission was calculated as:

$$n=-\log_{10}\left(B\right).$$

This formulation ensures that attenuation requirements remain independent of shielding material choice. Barrier thicknesses were computed using material‐specific attenuation parameters derived in Section 2.1. Because photon attenuation deviates from ideal exponential behavior due to spectral hardening and buildup effects, both the first and equilibrium TVLs were applied:

(1) $$\mathrm{Thicknes}\;s_{\mathrm{material}}=TVL_{1,\mathrm{material}}+\left(n-1\right)\cdot TVL_{e,\mathrm{material}}$$

To obtain the required thickness for candidate materials relative to NCRP‐151 compliant concrete, the attenuation requirement imposed by the concrete reference barrier was first determined:

$$n=\frac{\mathrm{Thicknes}s_{\mathrm{concrete}}-TVL_{1,\mathrm{concrete}}}{TVL_{e,\mathrm{concrete}}}+1$$

The corresponding thickness for each candidate material was then calculated using equation 1. This procedure preserves the required transmission factor:

$$\;B_{\mathrm{material}}=B_{\mathrm{concrete}}$$

thereby ensuring equivalent shielding performance across materials.

These calculated thicknesses were then implemented in the vault model to replace the conventional barriers, enabling a direct comparison of dose distributions and spatial requirements across materials. This approach ensured that each simulation reflected a clinically realistic barrier configuration while maintaining equivalent shielding performance criteria.

#### 2.2.2 Dose‐based thickness refinement

A certain amount of deviation is expected in the simulations of section 2.2.1 because narrow‐beam TVL‐based attenuation models do not fully capture the broad‐beam radiation transport encountered in a clinical vault. To account for this, a dose‐based refinement procedure was implemented. Barrier thicknesses were recalculated using Monte Carlo–scored dose ratios relative to the concrete reference configuration. For each candidate material, the correction term was calculated relative to the concrete reference configuration using:

$$\Delta \;t_{material}=TVL_{material}\cdot \log_{10}\left(\frac{D_{material}}{D_{concrete}}\right)$$

Where $D_{material}$ represents Monte Carlo–scored dose in the controlled area, $D_{concrete}$ is Monte Carlo–scored dose for NCRP‐151 compliant concrete, and $TVL_{material}$ is TVL_1_ derived in Section 2.1.

The refined barrier thickness was then obtained as:

$$\;t_{refined}=t_{preliminary}+\Delta t_{material}$$

### 2.3 Analysis of materials in LINAC vault geometry

#### 2.3.1 LINAC vault geometry

The simulation modeled a generic radiotherapy treatment room housing a medical LINAC with primary and secondary barriers, maze entrance, and ceiling/floor structures. The base vault concrete geometry was constructed to mimic a typical clinical layout: an inner room of approximately 8.36 m (length) × 12.06 m (width) × 2.90 m (height), surrounded by thick secondary wall of 1.40 m thickness. On each side of the wall, a 2.50 m thick primary barrier was designated to directly intercept the primary beam. An entry maze hallway with two right‐angle turns was included on the opposite side of the room to represent a typical maze‐type LINAC door configuration.^1^ The floor and ceiling were assumed to be 2.00 m of thick concrete, forming part of the foundation.^1^ Figure 1b illustrates the geometry and components of the simulated vault. For each candidate material, the primary and secondary barrier thicknesses were varied according to their experimental linear attenuation coefficients.

#### 2.3.2 LINAC beam setup in TOPAS

The LINAC beam was modeled after a conventional Siemens Oncor 6 MV and 18 MV photon beam.^9^ The inclusion of 18 MV represents a worst‐case scenario for shielding design, as this energy maximizes both photon penetration and photoneutron production (threshold ∼8 MeV for most nuclei). We deliberately selected 18 MV to provide a demanding comparison condition for evaluating high‐density composite materials. It should be noted that modern clinical practice increasingly favors 6 MV beams, which produce negligible photoneutrons and would require substantially less shielding than modeled here.^17^

To accurately simulate the radiation field, the LINAC head components were modeled in Geant4. The head geometry included a tungsten bremsstrahlung target struck by high‐energy electron beams, a primary collimator, a flattening filter, and adjustable jaws. To improve computational efficiency, a two‐step simulation strategy was employed. In the first step, a phase space was generated just above the treatment head by simulating 4 × 10^7^ primary 6 and 18 MeV electrons accordingly striking the target and yielding bremsstrahlung photons and secondary particles. This phase‐space file, containing detailed information on particle type, energy, direction, and position, effectively captured the emitted photon spectrum of the LINAC. In the second step, this phase space source was used as the input for shielding simulations within the vault geometry per material. The final vault simulations used an open, non‐MLC‐modulated 40 × 40 cm^2^ field at isocenter, with a source‐to‐surface distance (SSD) of 100 cm. A 36 × 36 × 36 cm^3^ water‐equivalent phantom was positioned at isocenter to represent patient scatter conditions. Two irradiation configurations were evaluated in the vault simulations: a full 360° rotational irradiation geometry and a conventional worst‐case single‐angle geometry toward a primary wall. In the rotational geometry, the gantry angle was incremented in 20° steps over a full 360° rotation for a total of 18 discrete orientations. Each orientation was assigned equal statistical weight, resulting in a uniform angular distribution of incident radiation. In the worst‐case primary‐barrier geometry, the 18 MV beam was directed perpendicularly toward the primary barrier and without a scattering phantom at isocenter, consistent with the conventional NCRP‐151 primary‐barrier evaluation approach. These results provide additional support for the proposed shielding configuration by demonstrating that the control‐room dose remains below the design goal under both the expected rotational‐use scenario and a conservative direct‐beam condition.

Radiation transport through the room and shielding was simulated with physics models suitable for high‐energy photons: electromagnetic processes up to 20 MeV were included. Photon interactions such as Compton scattering, pair production, and the photoelectric effect were enabled through the selected physics list. Photoneutron production was modeled using the G4PhotoNuclearProcess with appropriate cross‐sections for (γ,n) reactions in high‐Z materials.^18^

Several variance‐reduction techniques were applied to achieve efficient deep‐shielding simulations with acceptable statistical uncertainty. Bremsstrahlung photon splitting was employed (split factor of 1:300), biasing photon production in the target so that more photons with proportionally reduced statistical weight were directed toward the primary barrier. The 1:300 splitting factor was selected based on preliminary scaling simulations as a practical compromise between computational efficiency and statistical precision, with the goal of maintaining less than 5% statistical uncertainty at the controlled‐area scoring location while keeping the total CPU time within available computational resources. This increased the number of photons reaching and penetrating the shielding, improving tally precision behind thick barriers without requiring an impractically large number of primary histories. Statistical uncertainties below 5% at the primary measurement locations were measured by running three simulations on the concrete control, each with 10^8^ total particle histories. Because repeated uncertainty verification was limited to the concrete reference configurations, small differences among the candidate‐material dose values should be interpreted cautiously. The TOPAS input files used for the 6 MV and 18 MV vault simulations and TVL calculations are available in a public GitHub repository.^19^

#### 2.3.3 Dose scoring and attenuation parameters

Radiation detectors were positioned at critical locations to tally the dose and flux emerging from the shielding under each scenario. Key scoring locations included: (1) controlled room region 30 cm beyond the primary barrier on the outside wall surface,^1^ (2) at the entry maze door position in the maze corridor, 30 cm away from the door, and (3) at the isocenter inside the room for normalization and to record the unshielded dose as a reference. Figure 1b shows the diagram of the three key scoring locations. Detectors were modeled as either a small tissue‐equivalent volume or as mesh tallies recording particle fluence spectra. Total dose was scored using DoseToMedium and normalized to mSv/week using the stated weekly workload. For the 18 MV case, additional particle‐filtered DoseToMedium scorers were placed at the same primary controlled‐area scoring location to separately estimate gamma and neutron dose contributions using TOPAS particle filters for gamma and neutron. The neutron‐filtered scorer records energy deposition associated only with neutron tracks. Energy deposited by neutron‐induced secondary charged particles or photons is therefore excluded from this quantity, which should not be interpreted as the complete neutron ambient dose equivalent or total neutron‐attributable dose. This particle‐separated analysis was performed for ordinary concrete and representative high density composite materials. The scored doses were normalized to mSv/week assuming 180 patients/week (30 patients/day × 6 days/week) at 3 Gy per patient, yielding a workload of 540 Gy/week. This slightly exceeds the NCRP‐151 recommended workload of 500 Gy/week for high‐energy accelerators ^1^ and thus represents a conservative assumption. Because the same workload normalization was applied to all shielding materials, the comparative material ranking and relative thickness‐reduction conclusions are independent of the absolute workload assumption.

### 2.4 Clinical facility analysis

To evaluate the potential space‐efficiency implications of reduced barrier thickness, we analyzed the floor plan of a major academic medical center's radiation oncology department, anonymized for publication. The facility currently houses four LINAC vaults with conventional NCRP‐151–compliant shielding, consisting of 2.5 m primary barriers and 1.4 m secondary barriers. Using the material‐specific barrier thicknesses derived from the TVL‐based calculation and dose‐refinement procedure, we estimated the shielding‐related floor‐area reduction that could be achieved by replacing ordinary concrete with each candidate shielding material.

In all configurations, the internal treatment room dimensions were held constant to preserve equivalent clinical functionality, while exterior wall thicknesses were adjusted according to each material's attenuation properties. This analysis is intended to provide an illustrative estimate of potential space‐efficiency benefit.

## 3 RESULTS

### 3.1 Materials TVL and linear attenuation coefficients

The Monte Carlo–derived μ and TVLs are summarized in Table 2. Conventional concrete exhibited a linear attenuation coefficient of 0.08 cm^−1^ with TVL_1_ and TVL_2_ values of 21.1 cm and 27.5 cm for the 6 MV beam and 30.8 cm and 38.0 cm for the 18 MV beam, respectively. These values are consistent with the expected trend of increased photon penetration at higher beam energy and reflect attenuation behavior under the narrow‐beam configuration used in this study.^20^ The present narrow‐beam TVL_1_ values were compared with the small‐cone‐angle Monte Carlo results reported by Jaradat and Biggs. The simulated TVL_1_ values differed by 1.6 cm at 6 MV and 1.4 cm at 18 MV, corresponding to relative deviations of 8.2% and 4.8%, respectively, calculated using the Jaradat and Biggs values as the reference denominators.  The modest positive differences may be attributable to variations in concrete density, composition, and scoring geometries. In contrast, nanocomposite materials demonstrated markedly improved attenuation performance. Yb_2_O_3_ polymer yielded μ values of 0.24 cm^−1^ for both 6 MV and 18 MV beams, with corresponding TVL_1_ values of 6.5 cm and 9.4 cm, respectively. Datolite–Galena concrete yielded μ values of 0.22 cm^−1^ and 0.21 cm^−1^ for 6 MV and 18 MV beams, respectively, with corresponding TVL_1_ values of 8.6 cm and 11.0 cm. Steel–magnetite and magnetite–lead concretes also showed improved photon attenuation compared with conventional concrete, with TVL_1_ values of approximately 9–10 cm for 6 MV and 12–13 cm for 18 MV.

**TABLE 2:** Calculated Linear attenuation coefficient (μ), TVL1, TVL2, and TVLe of each material from the data obtained through Monte Carlo simulations of 6 MV and 18 MV photon transmission across a range of slab thicknesses for each material.

	6 MV				18 MV			
Material	μ	TVL_1_ (cm)	TVL_2_ (cm)	TVL_e_ (cm)	μ	TVL_1_ (cm)	TVL_2_ (cm)	TVL_e_ (cm)
Concrete	0.08	21.1	27.5	26.02	0.08	30.8	38.0	28.5
Bi_2_O_3_ Concrete	0.13	13.5	17.2	17.13	0.13	17.9	20.1	15.9
Magnetite–Lead Concrete	0.18	10.0	12.6	12.0	0.18	13.1	14.8	12.0
Steel–Magnetite Concrete	0.17	9.5	11.8	13.0	0.18	13.0	14.9	11.8
Datolite–Galena Concrete	0.22	8.6	10.9	11.0	0.21	11.0	12.1	10.0
Yb_2_O_3_ Polymer	0.24	6.5	8.4	9.4	0.24	9.4	11.6	8.0

### 3.2 Dose scoring and experimental wall thicknesses

Dose scoring at the controlled area 30 cm beyond the primary barrier revealed that standard concrete provided the lowest average dose, with a weekly value of 1.79 × 10^−2^ mSv/week and 2.45 × 10^−2^ mSv/week for 6 MV and 18 MV beams (Tables 3a and 3c). When the calculated thicknesses were applied to the candidate shielding materials, the resulting controlled‐area doses exhibited modest deviations relative to concrete. Most materials yielded slightly higher doses. These differences reflect the limitations of idealized TVL‐based attenuation models when applied to the complex radiation environment of a LINAC vault, where leakage radiation, scatter contributions, and spectral modifications influence the transmitted dose.

**TABLE 3:** Average controlled‐area doses and corresponding primary and secondary wall thicknesses obtained from preliminary and refined shielding calculations for 6 MV and 18 MV LINAC. Results are shown separately for the full 360° rotational configuration and the conventional worst‐case single‐angle primary‐barrier configuration. The worst‐case single‐angle configuration used a beam directed perpendicularly toward the primary barrier with a 40 × 40 cm^2^ open field and no scattering phantom at isocenter. The uncertainty shown for the concrete reference represents one run‐to‐run standard deviation from three simulations performed with independent random seeds. Equivalent repeat simulations were not performed for all candidate‐material configurations.

(a) 6 MV, Rotational configuration						
Material	Average dose in controlled area with the preliminary thicknesses (mSv/Week)	Primary wall thickness in preliminary calculation (m)	Secondary wall thickness in preliminary calculation (m)	Refined primary wall thickness (m)	Refined secondary wall thickness (m)	Average dose in controlled area with the refined thicknesses (mSv/Week)
Concrete	1.79 × 10^−2^	2.50	1.40	2.50	1.40	(1.79 ± 0.05) × 10^−2^
Bi_2_O_3_	1.86 × 10^−2^	1.64	0.92	1.65	0.92	1.79 × 10^−2^
Magnetite‐Lead	1.89 × 10^−2^	1.25	0.70	1.26	0.70	1.74 × 10^−2^
Steel‐Magnetite	1.80 × 10^−2^	1.23	0.70	1.24	0.70	1.76 × 10^−2^
Datolite‐Galena	1.92 × 10^−2^	1.03	0.58	1.04	0.58	1.76 × 10^−2^
Yb_2_O_3_ Polymer	1.92 × 10^−2^	0.83	0.47	0.84	0.47	1.79 × 10^−2^

(b) 6 MV, Worst‐case single‐angle configuration						
Material	Average dose in controlled area with the preliminary thicknesses (mSv/Week)	Primary wall thickness in preliminary calculation (m)	Secondary wall thickness in preliminary calculation (m)	Refined primary wall thickness (m)	Refined secondary wall thickness (m)	Average dose in controlled area with the refined thicknesses (mSv/Week)
Concrete	4.50 × 10^−2^	2.50	1.40	2.50	1.40	(4.50 ± 0.14) × 10^−2^
Bi_2_O_3_	4.63 × 10^−2^	1.64	0.92	1.65	0.92	4.48 × 10^−2^
Magnetite‐Lead	4.71 × 10^−2^	1.25	0.70	1.27	0.70	4.46 × 10^−2^
Steel‐Magnetite	4.60 × 10^−2^	1.23	0.70	1.24	0.70	4.50 × 10^−2^
Datolite‐Galena	4.82 × 10^−2^	1.03	0.58	1.05	0.58	4.48 × 10^−2^
Yb_2_O_3_ Polymer	4.81 × 10^−2^	0.83	0.47	0.85	0.47	4.45 × 10^−2^

(c) 18 MV, Rotational configuration						
Material	Average dose in controlled area with the preliminary thicknesses (mSv/Week)	Primary wall thickness in preliminary calculation (m)	Secondary wall thickness in preliminary calculation (m)	Refined primary wall thickness (m)	Refined secondary wall thickness (m)	Average dose in controlled area with the refined thicknesses (mSv/Week)
Concrete	2.45 × 10^−2^	2.50	1.40	2.50	1.40	(2.45 ± 0.10) × 10^−2^
Bi_2_O_3_	2.53 × 10^−2^	1.40	0.79	1.41	0.79	2.43 × 10^−2^
Magnetite‐Lead	3.86 × 10^−2^	1.05	0.59	1.08	0.61	2.40 × 10^−2^
Steel‐Magnetite	2.77 × 10^−2^	1.04	0.58	1.05	0.59	2.40 × 10^−2^
Datolite‐Galena	3.94 × 10^−2^	0.88	0.49	0.91	0.51	2.42 × 10^−2^
Yb_2_O_3_ Polymer	3.97 × 10^−2^	0.71	0.40	0.73	0.42	2.39 × 10^−2^

(d) 18 MV, Worst‐case single‐angle configuration						
Material	Average dose in controlled area with the preliminary thicknesses (mSv/Week)	Primary all thickness in preliminary calculation (m)	Secondary wall thickness in preliminary calculation (m)	Refined primary wall thickness (m)	Refined secondary wall thickness (m)	Average dose in controlled area with the refined thicknesses (mSv/Week)
Concrete	6.28 × 10^−2^	2.50	1.40	2.50	1.40	(6.28 ± 0.25) × 10^−2^
Bi_2_O_3_	6.40 × 10^−2^	1.40	0.79	1.41	0.79	6.20 × 10^−2^
Magnetite‐Lead	6.93 × 10^−2^	1.05	0.59	1.07	0.61	6.19 × 10^−2^
Steel‐Magnetite	6.79 × 10^−2^	1.04	0.58	1.06	0.59	6.11 × 10^−2^
Datolite‐Galena	7.11 × 10^−2^	0.88	0.49	0.91	0.51	6.23 × 10^−2^
Yb_2_O_3_ Polymer	7.28 × 10^−2^	0.71	0.40	0.74	0.42	6.26 × 10^−2^

Following the thickness refinement, all candidate shielding materials exhibited dose values closely matching the concrete reference case. The resulting controlled‐area doses were within about 10% relative to standard concrete as seen in Table 3, indicating strong convergence toward transmission‐equivalent shielding performance. At the maze‐exit scoring location, no energy‐deposition events were recorded across repeated simulation runs for any evaluated material. Consequently, the scored maze‐exit dose was zero within the statistical sensitivity of the simulations.

All candidate materials satisfied the NCRP‐151 design goal of 0.1 mSv/week for controlled areas.^1^ Under the worst‐case single‐angle configuration, the refined controlled‐area doses ranged from 4.45 × 10^−2^ to 4.50 × 10^−2^ mSv/week for the 6 MV beam and from 6.11 × 10^−2^ to 6.28 × 10^−2^ mSv/week for the 18 MV beam (Table 3). Although the worst‐case single‐angle configuration produced higher controlled‐area doses than the 360° rotational geometry, all values remained below the weekly design goal after thickness refinement. These results provide a conservative benchmark for the proposed shielding configurations and demonstrate that the evaluated materials maintain the required controlled‐area protection level even under direct primary‐beam incidence.

Table 4 shows the neutron dose component separated from the total dose using particle‐specific scoring. The neutron dose ranged from 1.34 × 10^−5^ to 2.75 × 10^−5^ mSv/week, indicating that the neutron contribution was negligible under the 18 MV worst‐case single‐angle irradiation condition.

**TABLE 4:** Total dose and Neutron dose components at the primary controlled‐area scoring location for the 18 MV worst‐case configuration.

Material	Total dose (mSv/Week)	Neutron dose (mSv/Week)
Concrete	(6.28 ± 0.25) × 10^−2^	(1.37 ± 0.11) × 10^−5^
Bi_2_O_3_	6.20 × 10^−2^	1.43 × 10^−5^
Magnetite‐Lead	6.19 × 10^−2^	2.75 × 10^−5^
Steel‐Magnetite	6.11 × 10^−2^	2.64 × 10^−5^
Datolite‐Galena	6.23 × 10^−2^	2.73 × 10^−5^
Yb_2_O_3_ Polymer	6.26 × 10^−2^	1.34 × 10^−5^

### 3.3 Space efficiency

**FIGURE 2:**
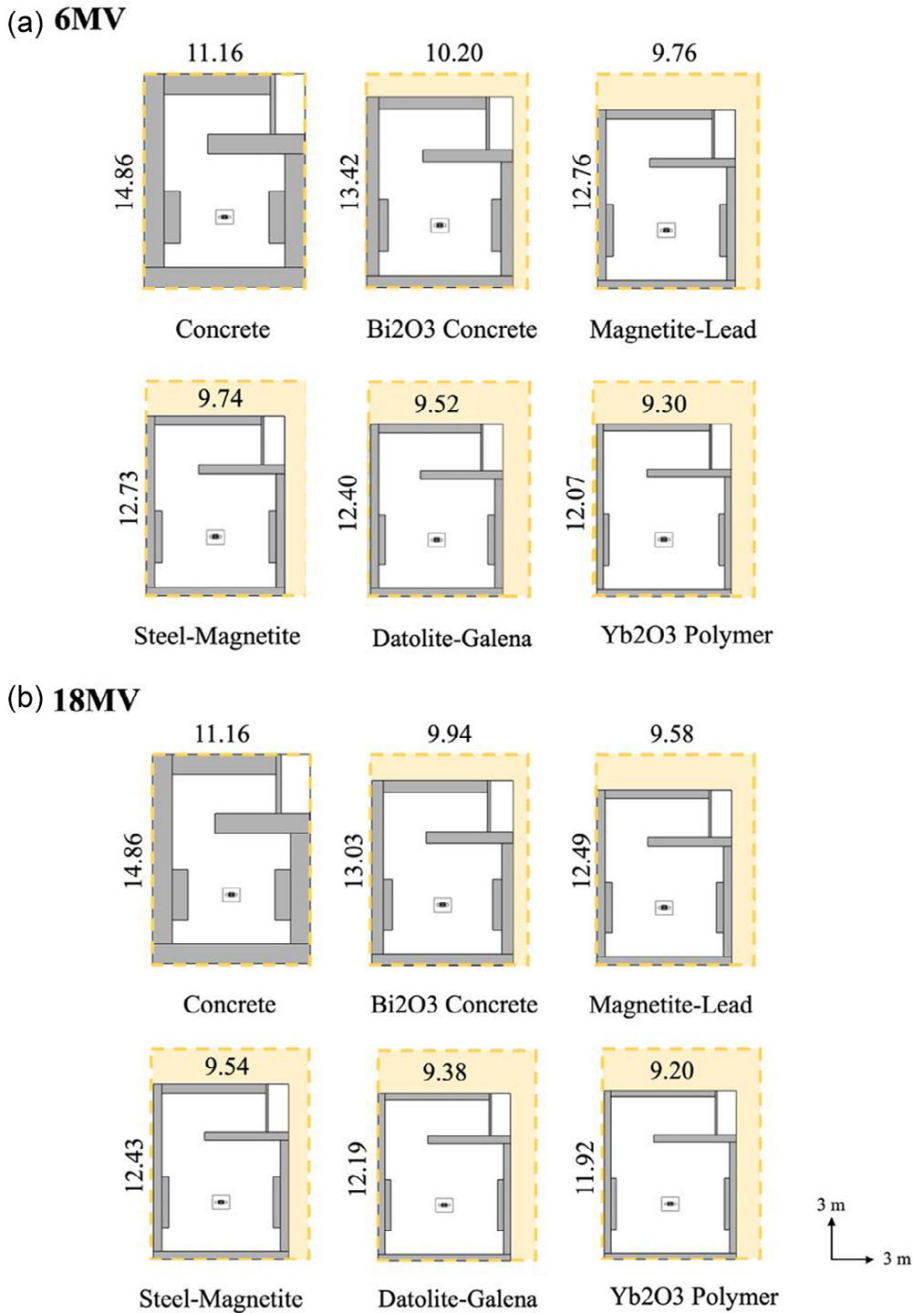
Floor plan layouts of LINAC vaults constructed using different candidate shielding materials. Each diagram reflects the wall thickness required to meet NCRP dose limits for a 6 MV and an 18 MV LINAC. The dashed outline represents the footprint of the conventional concrete design for direct spatial comparison. Annotated dimensions are given in meters. A 3 m × 3 m scale bar is provided for reference.

Barrier thickness requirements derived from TVL data translated into substantial floor‐area savings for the selected high‐density composite materials. As shown in Figure 2, each shielding material produces a distinct vault footprint due to differences in required wall thickness. Compared to the baseline of 165.8 m^2^ for concrete, the five candidate materials achieved 17%–32% reductions for 6 MV beams and 22%–34% reductions for 18 MV beams in required footprint as seen in Table 5.

**TABLE 5:** Required floor area, and relative floor‐area reduction for each candidate shielding material, listed in order of increasing area reduction.

(a) 6 MV		
Material	Required Floor Area (m^2^)	Floor Area Reduction (%)
Concrete	165.84	0.00
Bi_2_O_3_ Concrete	136.88	17.46
Magnetite‐Lead	124.54	24.90
Steel‐Magnetite	123.99	25.23
Datolite‐Galena	118.05	28.81
Yb_2_O_3_ Polymer	112.25	32.31

18 MV		
Material	Required Floor Area (m^2^)	Floor Area Reduction (%)
Concrete	165.84	0.00
Bi_2_O_3_	129.52	21.90
Magnetite‐Lead	119.65	27.85
Steel‐Magnetite	118.58	28.50
Datolite‐Galena	114.34	31.05
Yb_2_O_3_ Polymer	109.66	33.88

## 4 DISCUSSION

The present study shows that high‐density composite shielding materials can substantially reduce shielding‐related vault footprint while maintaining controlled‐area doses below the NCRP‐151 design goal. Compared with conventional concrete, the five candidate materials achieved calculated floor‐area reductions of 17%–32% for 6 MV beams and 22%–34% for 18 MV beams. After dose‐based thickness refinement, all evaluated materials remained below the 0.1 mSv/week controlled‐area design goal under both the 360° rotational geometry and the worst‐case single‐angle primary‐barrier configuration. From a clinical planning perspective, these space savings could be meaningful in capacity‐constrained radiotherapy facilities. For example, assuming each LINAC treats approximately 30 patients per day over 250 treatment days per year,^8^ a facility layout that ultimately enables the addition of one treatment unit could correspond to approximately 7,500 fractions or 1,500–2,000 additional patients annually, assuming a mix of conventional, hypofractionated, and SBRT regimens with an average of approximately 4 fractions per course.

The performance differences among high‐density composite materials can be attributed to the complex interplay between photon attenuation and secondary particle production. High‐Z additives enhance gamma attenuation through increased photoelectric and pair production cross‐sections, enabling thinner barriers. At 18 MV, photoneutron production may also occur in high‐Z materials. However, these values remained negligible relative to the total controlled‐area dose, ranging from 1.34 × 10^−5^ to 2.75 × 10^−5^ mSv/week under the 18 MV worst‐case single‐angle condition. Their very small magnitude is consistent with substantial neutron attenuation by the hydrogen‐containing concrete and composite barriers at the modeled thicknesses and confirms that the total controlled‐area doses reported in Table 3 were dominated by the photon component.

The 6 MV results should be interpreted as an upper‐bound comparison for lower‐energy shielding behavior relative to expected 6FFF performance. Existing 6FFF shielding studies, including Kry et al., have reported reduced shielding requirements for FFF beams compared with conventional flattened beams.^2^ Therefore, a true 6FFF configuration would be expected to require equal or less shielding than the modeled flattened 6 MV case, with reported reductions on the order of approximately 10%–20%, depending on beam model, field size, and scattering angle.

The results highlight the potential role of shielding material selection within the existing NCRP‐151 framework. The NCRP‐151 design goal of 0.1 mSv/week for controlled areas already incorporates substantial safety margins. The goal corresponds to an annual dose of approximately 5 mSv, well below the NCRP‐recommended occupational dose limit of 50 mSv/year.^1^ Our finding that multiple candidate materials can achieve the same dose‐based shielding benchmark with substantially reduced physical barrier thickness suggests that material selection may provide a pathway to improve space efficiency while preserving established shielding design goals.

Several challenges must be addressed before clinical implementation. While advanced composites offer substantial shielding benefits, their wider adoption will likely depend on cost and constructability. Ordinary concrete remains the dominant shielding material not only because of its radiological adequacy, but also because it is widely available and comparatively cost‐effective for large infrastructure applications.^21^ At present, the most realistic pathway to broader clinical use may be concrete‐compatible high‐density mixtures such as Bi_2_O_3_ concrete, Datolite‐Galena concrete, and Steel‐Magnetite concrete,^22^ particularly if they can deliver meaningful floor‐area savings for only a modest increase in material cost. Supporting this view, a recent life‐cycle study of shielding concrete found magnetite‐based heavyweight concrete to have approximately 23% lower life‐cycle cost than barite‐based alternatives while still satisfying shielding and mechanical requirements.^23^ By contrast, Yb_2_O_3_ polymer achieved some of the greatest footprint reductions, but, while direct quantitative cost data for Yb_2_O_3_ polymer shielding materials are limited, its rare‐earth oxide content and polymer‐based formulation would likely place it in a substantially higher cost tier than ordinary concrete or concrete‐compatible high‐density mixtures.^14^, ^24^

The attenuation coefficients and TVL parameters employed in this study were derived under narrow‐beam conditions, consistent with standard shielding data reported in the literature.^25^ While narrow‐beam geometry provides well‐defined exponential attenuation behavior and enables material‐to‐material comparison, it does not fully represent the radiation transport characteristics of a clinical treatment vault. In broad‐beam environments, scattered radiation and buildup effects contribute to the transmitted dose, generally resulting in reduced effective attenuation and therefore larger shielding requirements. Consequently, barrier thicknesses estimated solely from narrow‐beam TVL data may underestimate the shielding needed under realistic vault conditions. To address this limitation, a dose‐based refinement framework was implemented, effectively introducing an equivalent thickness correction that accounts for leakage radiation, scatter contributions, and spectral modifications captured by Monte Carlo simulation. This approach preserves the analytical advantages of narrow‐beam attenuation models while compensating for their known limitations in broad‐beam geometries.

Future research should prioritize experimental validation using anthropomorphic phantoms and real beam measurements to confirm our Monte Carlo predictions. The development of standardized testing protocols for advanced shielding materials will be essential for regulatory acceptance. Finally, comprehensive economic modeling that incorporates construction costs, operational savings, and the revenue potential of increased treatment capacity will help justify the initial investment in these advanced materials.

## 5 CONCLUSION

This Monte Carlo study provides a systematic computational comparison of alternative high‐density composite materials for space‐efficient radiotherapy vault design. Under the modeled assumptions, selected materials achieved the same dose‐based shielding objective as ordinary concrete with reduced physical barrier thickness. All evaluated materials met the NCRP‐151 controlled‐area design goal under both rotational and worst‐case single‐angle irradiation conditions. These findings suggest that material selection may provide a practical pathway to improve shielding‐related space efficiency. Although practical challenges such as material costs and regulatory inertia remain, the possibility of expanding treatment availability warrants thoughtful consideration. As accelerator technology and materials science continue to advance, periodic review of established shielding design guidelines may help ensure that facility standards reflect the current stage of knowledge while maintaining appropriate safety margins. The central question may not be simply whether shielding requirements can be reduced, but how best to balance safety, practicality, and equitable patient access to life‐saving treatment in future design standards.

## AUTHOR CONTRIBUTIONS

Beechui Koo contributed to simulation, supercomputing, and manuscript writing. Richard Xu contributed to the simulation draft and shielding calculation. Morgan Glennie contributed to simulation validation and manuscript writing. Jungwook Shin contributed to simulation consultation and supervision. Karl Farrey contributed to shielding calculation validation and manuscript review. Siyong Kim contributed to simulation review and writing proofread. James Sohn contributed to study conception and overall operational verification.

## CONFLICT OF INTEREST STATEMENT

The authors declare no conflicts of interest.

## DATA AND AVAILABILITY STATEMENT

The TOPAS simulation input files used in this study are publicly available in the GitHub repository: https://github.com/koobcbc/Shielding‐TOPAS.
